# Development of a Nomogram to Predict 28-Day Mortality of Patients With Sepsis-Induced Coagulopathy: An Analysis of the MIMIC-III Database

**DOI:** 10.3389/fmed.2021.661710

**Published:** 2021-04-06

**Authors:** Zongqing Lu, Jin Zhang, Jianchao Hong, Jiatian Wu, Yu Liu, Wenyan Xiao, Tianfeng Hua, Min Yang

**Affiliations:** ^1^The 2nd Department of Intensive Care Unit, The Second Affiliated Hospital of Anhui Medical University, Hefei, China; ^2^The Laboratory of Cardiopulmonary Resuscitation and Critical Care Medicine, The Second Affiliated Hospital of Anhui Medical University, Hefei, China; ^3^Key Laboratory of Intelligent Computing & Signal Processing, Ministry of Education, Anhui University, Hefei, China

**Keywords:** sepsis-induced coagulopathy, logistic regression, short-time mortality, nomogram, MIMIC-III database, prediction of prognosis

## Abstract

**Background:** Sepsis-induced coagulopathy (SIC) is a common cause for inducing poor prognosis of critically ill patients in intensive care unit (ICU). However, currently there are no tools specifically designed for assessing short-term mortality in SIC patients. This study aimed to develop a practical nomogram to predict the risk of 28-day mortality in SIC patients.

**Methods:** In this retrospective cohort study, we extracted patients from the Medical Information Mart for Intensive Care III (MIMIC-III) database. Sepsis was defined based on Sepsis 3.0 criteria and SIC based on Toshiaki Iba's criteria. Kaplan–Meier curves were plotted to compare the short survival time between SIC and non-SIC patients. Afterward, only SIC cohort was randomly divided into training or validation set. We employed univariate logistic regression and stepwise multivariate analysis to select predictive features. The proposed nomogram was developed based on multivariate logistic regression model, and the discrimination and calibration were verified by internal validation. We then compared model discrimination with other traditional severity scores and machine learning models.

**Results:** 9432 sepsis patients in MIMIC III were enrolled, in which 3280 (34.8%) patients were diagnosed as SIC during the first ICU admission. SIC was independently associated with the 7- and 28-day mortality of ICU patients. K–M curve indicated a significant difference in 7-day (Log-Rank: *P* < 0.001 and *P* = 0.017) and 28-day survival (Log-Rank: *P* < 0.001 and *P* < 0.001) between SIC and non-SIC groups whether the propensity score match (PSM) was balanced or not. For nomogram development, a total of thirteen variables of 3,280 SIC patients were enrolled. When predicted the risk of 28-day mortality, the nomogram performed a good discrimination in training and validation sets (AUROC: 0.78 and 0.81). The AUROC values were 0.80, 0.81, 0.71, 0.70, 0.74, and 0.60 for random forest, support vector machine, sequential organ failure assessment (SOFA) score, logistic organ dysfunction score (LODS), simplified acute physiology II score (SAPS II) and SIC score, respectively, in validation set. And the nomogram calibration slope was 0.91, the Brier value was 0.15. As presented by the decision curve analyses, the nomogram always obtained more net benefit when compared with other severity scores.

**Conclusions:** SIC is independently related to the short-term mortality of ICU patients. The nomogram achieved an optimal prediction of 28-day mortality in SIC patient, which can lead to a better prognostics assessment. However, the discriminative ability of the nomogram requires validation in external cohorts to further improve generalizability.

## Introduction

Sepsis, defined as a dysregulated host response to infection by the Surviving Sepsis Campaign 2016 guideline, remains the leading cause of life-threatening organ dysfunction in the intensive care unit (ICU) (1). Sepsis is rapidly becoming a significant global health burden. The World Health Organization declared that the mortality of hospital-treated adult patients with sepsis is ~189 per 100,000 person-years, and such a rate has been reported in up to 42% or even higher of ICUs depending on its severity in patients (2).

Coagulation abnormalities, as a severe complication, occur in almost all sepsis patients (3). The clinical manifestations of such abnormalities range from thrombocytopenia during the initial phase to advanced disseminated intravascular coagulation, with the latter always leading to multiple organ dysfunction syndromes (MODS) and indicates higher mortality (4). Coagulation abnormality in sepsis patients with a increased international normalized ratio (INR) and reduced platelet count is termed sepsis-induced coagulopathy (SIC) (5). Previous multicenter retrospective observational trials demonstrated that SIC is significantly associated with poor prognosis (6–8). Because SIC is a dynamic process, applying specific interventions based on stratifying SIC patients according to their mortality risks would provide improved strategies to prevent MODS. However, methods to calculate the mortality probability are rarely applied in clinical practice.

Recently, using the logistic regression model, a retrospective analysis of a nationwide study in Japan developed a SIC scoring system in which the platelet count, prothrombin time (PT)-INR and sequential organ failure assessment (SOFA) scores are associated with the 28-day mortality level of sepsis patients (9). Subsequent clinical investigations have shown the value of the SIC score system, for example, with a higher sensitivity (~84.4–96.1) in the prediction of the 28-day mortality of SIC patients compared with the International Society on Thrombosis and Haemostasis (ISTH) scoring system (10). Conversely, another published study demonstrated a smaller area under the curve (AUC) of the SIC system (~0.658) in predicting ICU mortality when compared with the SOFA, Acute Physiologic And Chronic Health Evaluation II (APACHE II) and ISTH scores (11). Therefore, the performances of the SIC scoring system in predicting the prognosis of SIC patients are inconsistent. Furthermore, because the highest total points of the SIC scoring system is six, the correlation between such points and critical patients' outcomes may be ambiguous. Because of the suboptimal performance of existing methods, it is necessary to develop a novel prediction model for the subgroup combined with SIC.

The nomogram as a visualization tool has been widely used in clinical prognosis research on critical patient and cancer patient survival studies (12–14). The primary aim of the present study is to develop a novel prediction nomogram for the 28-day mortality risk in SIC patients. The secondary aim is to explore the differences in the clinical characteristics between SIC and non-SIC patients, and verify whether SIC poses a short-term mortality risk for patients in the ICU.

## Methods

### Source of Data

An open and free critical care database, which contained comprehensive clinical data of patients admitted to the Beth Israel Deaconess Medical Center in Boston, Massachusetts between June 2001 and October 2012, termed the Medical Information Mart for Intensive Care (MIMIC)-III v 1.4, was retrieved (15). This database was released on 2nd September 2016, in which extensive and de-identified in-hospital information of over 40,000 patients was included. All data were classified into 26 tables, consisting of demographic characteristics, vital signs, laboratory test results, imaging examinations, and a data dictionary. Included patients were assigned a special code on each hospital and ICU admission, thus we could relate each table using these codes to obtain a complete hospitalization record. Hospital staff entered the final precise diagnosis according to the International Classification of Disease 9th Edition code when patients were discharged. In the present study included datasets were extracted by Lu, who had completed the collaborative institution training initiative program course (Record ID: 36763801). Because the present study was conducted using an anonymized public database that satisfied review committee agreements, the requirement for ethical consent was not necessary. Rather, the TRIPOD statement was applied in the present study (16).

### Study Population and Data Extraction

#### Sepsis

The following data were extracted from the MIMIC-III database: (1) demographic data; (2) first care unit; (3) outcomes, including ICU stay time, 7-day mortality, 28-day mortality, hospital mortality; (4) severity score, including SOFA and logistic organ dysfunction (LODS) score; (5) mean value of vital signs and the poorest laboratory test value during the first day after ICU admission; (6) infectious sites defined using PgAdmin software (version 4.1, Bedford, MA, USA). We retrieved adult sepsis patients (≥18 years) as defined according to the Sepsis-3.0 criterion: (1) existing evidence of suspected or confirmed infection; (2) SOFA score ≥2 (17). Exclusion criteria were: (1) age <18 years; (2) pregnant women; (3) patients with congenital coagulopathy; (4) the coagulation function was frequently affected by the pathologic states of tumors and the chemotherapy agent used, thus patients with various cancer types were excluded; (5) patients who died or were discharged within 24 h after ICU admission (Supplementary Figure 1).

#### Sepsis-Induced Coagulopathy

On the basis of all eligible sepsis patients, SIC patients were defined as fulfilling the Toshiaki Iba's criteria, also referred to as the Sepsis-induced coagulopathy scoring system (9). Patients were considered to display SIC when having a total SIC score ≥4 with a total score of PT-INR and platelet count parameters >2 during the first day of ICU admission. Afterwards, the parameters of the eligible SIC patients were applied in the logistic regression to construct the proposed prediction model. The flowchart of study design and data extraction can be found in Supplementary Figure 1.

### Statistical Analysis

Normal distributions were confirmed by Agostino tests. Continuous variables are presented as the mean (standard deviation) for parametric variables and as the median (interquartile ranges) for non-parametric variables. Continuous variables were compared by unpaired Student's test or Mann–Whitney *U*-test. Categorical variables were compared using the χ^2^-test or Fisher exact test.

Both, the 7- and 28-day survival curves were generated using the Kaplan–Meier method and compared by the log-rank test. To resolve the baseline imbalance problem, the sample was performed using the propensity score match (PSM), and we further explored the difference in short survival time between the SIC and non-SIC patients.

Prior to construction of the nomogram, only SIC patients were randomly assigned to the training or validation cohort based on a ratio of 7:3. In the training cohort, all significant variables associated with the 28-day mortality through univariate logistic regression analysis were candidates for stepwise multivariate analysis. Although these variables were clinically associated with the 28-day mortality, they were not statistically significant; however, they were still included. Besides, those categorical variables in which a set of meaningful values existed were also included. The variance inflation factor (VIF) was calculated to detect the potential collinearity between continuous variables. When the arithmetic square root of the VIF was >2, collinearity was considered to exist and it will be solved by regularization. Stepwise backward regression was conducted according to the Akaike information criterion (AIC), and the best model should achieve a minimum AIC value. Subsequently, the nomogram was plotted using the “rms” package of R software based on the results of multivariate logistic regression. Finally, the predictive performance of the nomogram was evaluated using a calibration with 1,000 bootstrap resampling, and measured using the C-index.

For the clinical use of this model, both receiver operating characteristic (ROC) and decision curve analysis (DCA) were conducted to compare the performance of the SOFA, LODS, SAPS II, and SIC scores with the nomogram. The integrated discrimination improvement (IDI) and net reclassification improvement (NRI) indices of each clinical severity scoring system were also calculated. Furthermore, other common machine-learning models, including random forests (RF) and the support vector machine (SVM), were constructed to compare the generalizability and accuracy of each model.

All statistical analyses were performed using STATA 15.1 (College Station, Texas) and R 3.6.2 (Chicago, Illinois) software. Missing values were handled by the RF method, based on the “randomForest” package of R. However, these variables were omitted when >30% of the values were lacking. *P* < 0.05 was considered to indicate statistical significance.

## Results

### Characteristics of Included Sepsis Participants

A total of 9,432 sepsis patients were included, of whom 34.8% were SIC patients. The baseline characteristics are listed in Table 1. The SIC patients with a median age of 67 (54, 79) years were younger than the non-SIC patients of 72 (58, 82) years. Regarding comorbidity, we unexpectedly found that the SIC patients were less likely to suffer from hypertension, chronic obstructive pulmonary disease (COPD), diabetes and myocardial infarction, but not liver disease, when compared with the non-SIC patients. However, the SIC patients displayed higher lactate-max, creatinine-max, and blood urea nitrogen-max levels, INR-max, PT-max, mean corpuscular volume-min (MCV-min), and red cell distribution width-max (RDW-max) and lower platelet levels, PO_2_-min as well as serum PH-min value in the first 24 h since ICU admission. Additionally, there was a statistical difference in the length of the ICU stay (*P* < 0.001), 7-day (*P* < 0.001), 28-day (*P* < 0.001), and hospital mortalities (*P* < 0.001) between the SIC and non-SIC patients, and the SIC patients had a higher critical illness score, including the SOFA, LODS and SAPS II. Finally, the SIC patients exhibited a higher frequency of epinephrine and/or norepinephrine administration.

**Table 1:** The characteristics of included patients when first ICU admission.

**Variables**	**All patients (*n* = 9432)**	**Non-SIC patients (*n* = 6152)**	**SIC patients (*n* = 3280)**	***p***
**Gender**, ***n*** **(%)**				<0.001
Male	5,070 (54)	3,111 (51)	1,959 (60)	
Female	4,362 (46)	3,041 (49)	1,321 (40)	
Age, years	69.90 (56.38, 80.85)	71.54 (58.18, 81.86)	66.93 (53.52, 79.08)	<0.001
**First care unit**, ***n*** **(%)**				<0.001
CCU	1,229 (13)	914 (15)	315 (10)	
CSRU	813 (9)	419 (7)	394 (12)	
MICU	5,158 (55)	3,324 (54)	1,834 (56)	
SICU	1,323 (14)	885 (14)	438 (13)	
TSICU	909 (10)	610 (10)	299 (9)	
**Outcome**				
ICU stay time, days	4.04 (1.92, 9.25)	3.92 (1.92, 8.92)	4.21 (1.96, 10.04)	<0.001
7-day mortality, *n* (%)	1,332 (14)	756 (12)	576 (18)	<0.001
28-day mortality, *n* (%)	2,669 (28)	1,555 (25)	1,114 (34)	<0.001
Hospital mortality, *n* (%)	2,452 (26)	1,380 (22)	1,072 (33)	<0.001
**Comorbidity**, ***n*** **(%)**				
Hypertension, *n* (%)	3,388 (36)	2,348 (38)	1,040 (32)	<0.001
COPD, *n* (%)	446 (5)	376 (6)	70 (2)	<0.001
Diabetes, *n* (%)	2,819 (30)	1,949 (32)	870 (27)	<0.001
MI, *n* (%)	320 (3)	238 (4)	82 (2)	<0.001
CHF, *n* (%)	316 (3)	225 (4)	91 (3)	0.027
Cardiac arrhythmias, *n* (%)	3,317 (35)	2,193 (36)	1,124 (34)	0.189
Liver disease, *n* (%)	1,118 (12)	338 (5)	780 (24)	<0.001
**Severity score**				
SOFA	5.00 (4.00, 8.00)	5.00 (3.00, 7.00)	7.00 (5.00, 10.00)	<0.001
LODS	5.00 (3.00, 7.00)	5.00 (3.00, 7.00)	6.00 (4.00, 8.00)	<0.001
SAPS II	42.00 (33.00, 52.00)	41.00 (32.00, 51.00)	44.00 (35.00, 55.00)	<0.001
**Vital signs^a^**				
Mean heartrate, (min^−1^)	87.49 (77.33, 98.93)	86.82 (76.50, 97.85)	88.93 (79.08, 101.47)	<0.001
MAP, (mmHg)	75.03 (68.81, 81.22)	75.48 (69.05, 81.83)	74.18 (68.29, 80.07)	<0.001
Mean resprate, (min^−1^)	19.56 (17.10, 22.42)	19.54 (17.17, 22.29)	19.62 (16.95, 22.75)	0.528
Mean temperature, (°C)	36.86 (36.44, 37.28)	36.88 (36.47, 37.29)	36.83 (36.40, 37.25)	<0.001
**Laboratory tests^b^**				
Mean glucose, (mg/dl)	137.50 (115.00, 161.67)	138.40 (116.00, 163.50)	135.27 (112.49, 158.76)	<0.001
Aniongap_max,	16.00 (14.00, 19.00)	16.00 (14.00, 19.00)	16.00 (14.00, 20.00)	0.133
Bicarbonate_min, (mEq/L)	21.00 (18.00, 24.00)	22.00 (19.00, 25.00)	20.00 (17.00, 23.00)	<0.001
Chloride_max, (mEq/L)	107.00 (103.00, 112.00)	107.00 (103.00, 111.00)	109.00 (104.00, 113.00)	<0.001
Hematocrit_min, (%)	29.00 (25.30, 33.30)	30.00 (26.70, 34.10)	26.80 (23.00, 31.10)	<0.001
Hemoglobin_min, (g/dL)	9.80 (8.50, 11.20)	10.10 (8.90, 11.50)	9.10 (7.90, 10.60)	<0.001
Lactate_max, (mmol/L)	2.63 (1.80, 3.60)	2.50 (1.70, 3.16)	3.00 (2.20, 4.80)	<0.001
Lowest platelet level, (K/uL)	176.00 (112.00, 247.00)	221.00 (179.00, 289.00)	93.00 (60.00, 121.00)	<0.001
Potassium_max, (K/uL)	4.50 (4.10, 5.10)	4.50 (4.10, 5.10)	4.60 (4.10, 5.30)	<0.001
PTT_max, (s)	36.10 (28.90, 48.80)	33.20 (27.60, 44.00)	40.70 (32.90, 58.82)	<0.001
INR_max,	1.40 (1.20, 1.80)	1.30 (1.20, 1.60)	1.64 (1.40, 2.20)	<0.001
PT_max, (s)	15.31 (13.70, 18.40)	14.60 (13.30, 16.80)	16.90 (15.00, 21.00)	<0.001
Sodium_min, (mEq/L)	137.00 (134.00, 140.00)	137.00 (134.00, 140.00)	136.00 (133.00, 139.00)	<0.001
BUN_max, (mg/dL)	28.00 (18.00, 47.00)	28.00 (18.00, 45.00)	30.50 (19.00, 50.00)	<0.001
WBC_max, (K/uL)	13.40 (9.40, 18.70)	14.00 (10.20, 19.30)	11.90 (7.70, 17.60)	<0.001
Po2-min, (mmHg)	89.34 (68.00, 104.06)	91.00 (70.00, 105.05)	86.48 (67.00, 102.12)	<0.001
Pco2-max, (mmHg)	46.08 (40.00, 51.00)	46.95 (40.00, 51.11)	45.45 (39.00, 50.00)	<0.001
PH-min	7.31 (7.26, 7.37)	7.32 (7.27, 7.37)	7.31 (7.23, 7.36)	<0.001
MCH_min, (pg)	30.10 (28.80, 31.50)	29.90 (28.50, 31.13)	30.50 (29.30, 32.10)	<0.001
MCHC_min, (g/L)	33.30 (32.20, 34.20)	33.10 (32.10, 34.00)	33.50 (32.40, 34.50)	<0.001
RDW_max, (%)	16.41 (15.32, 17.29)	15.45 (14.76, 16.77)	17.41 (16.30, 18.87)	<0.001
MCV_min, (fL)	90.00 (86.00, 94.00)	89.00 (86.00, 93.00)	90.00 (86.00, 95.00)	<0.001
Creatinine_max, (μmol/L)	114.92 (79.56, 194.48)	114.92 (79.56, 185.64)	123.76 (88.40, 212.16)	<0.001
**Infection site**, ***n*** **(%)**				
Lung, *n* (%)	3,440 (36)	2,355 (38)	1,085 (33)	<0.001
Urea, *n* (%)	2,807 (30)	1,923 (31)	884 (27)	<0.001
Catheter, *n* (%)	240 (3)	153 (2)	87 (3)	0.676
Bacteremia, *n* (%)	612 (6)	372 (6)	240 (7)	0.019
Septicemic, *n* (%)	120 (1)	72 (1)	48 (1)	0.266
**Treatment measures**				
MV, *n* (%)	2,493 (26)	1,639 (27)	854 (26)	0.542
Epinephrine, *n* (%)	329 (3)	156 (3)	173 (5)	<0.001
Norepinephrine, *n* (%)	2,076 (22)	1,219 (20)	857 (26)	<0.001

*Categorical data were presented as frequency (percentage), parametric continuous data were presented as median (interquartile ranges), whereas non-parametric continuous data were presented as median (interquartile ranges)*.

a *Vital signs were calculated as mean value during the first 24 h since ICU admission of each included patients*.

b *The laboratory tests recorded the worest value during the first 24 h since ICU admission of each included patients*.

*CCU, coronary care unit; CSRU, cardiac surgical intensive care unit; MICU, medical intensive care unit; SICU, surgical intensive care unit, TSICU, trauma/surgical intensive care unit; SOFA, Sequential Organ Failure Assessment; LODS, Logistic Organ Dysfunction System; SAPS II, Simplified acute physiology II; SAPS II, Simplified acute physiology; PT, Prothrombin Time; PTT, Partial Thromboplastin Time; INR, International Normalized Ratio; RDW, Red Blood Cell Distribution Widths; MV, Mechanical Ventilation; MAP, Mean arterial pressure*.

### SIC Was Independently Associated With the 7-day and 28-day Mortalities of Sepsis Patients

The result of multivariate logistic regression showed that SIC was an independent risk factor for the 7- and 28-day mortalities of the included patients, with an adjusted odds ratio of 1.52 [95% confidence interval (CI): 1.35, 1.71] and 1.52 (95% CI: 1.39, 1.67), respectively, after adjusting for baseline characteristics, vital signs, critical illness score, infection sites, and treatment measures. Subsequently, we conducted a PSM between the SIC and non-SIC cohorts according to the differences in the vital signs, critical illness score, infection sites, treatment measures and comorbidities in first 24 h since ICU admission. Kaplan–Meier's survival analysis found significant differences between the SIC and non-SIC patients in the 7- and 28-day survival whether or not a PSM was performed (Supplementary Figures 2, 3).

### Development of a Prediction Nomogram

Only 3,280 SIC patients were randomly assigned to the training (2,293 patients) or validation sets (987 patients). The data of non-SIC patients were not suitable for subsequent model development, since the model was designed to predict the short-term death risk in SIC patients. All variables of the included participants in each set are presented in Supplementary Table 1. No statistical differences in all the variables were found between the training and validation sets, except for the creatinine-max. The results of the univariate logistic analysis using the training cohort are presented in Table 2.

**Table 2:** Factors independently associated with 28-day mortality of patients with SIC by univariate logistic regression analysis in training cohort.

**Variables**	**OR (95% CI)**	***p*-value**
Age, y	1.01 (1.00, 1.02)	<0.001
Liver-disease, yes vs. no	1.58 (1.30, 1.93)	<0.001
Cirrhosis, yes vs. no	1.68 (1.28, 2.20)	<0.001
Mean heart rate (min-1)	1.02 (1.01, 1.02)	<0.001
MAP (mmHg)	0.97 (0.96, 0.98)	<0.001
Mean respiratory rate (min^−1^)	1.08 (1.06, 1.10)	<0.001
Mean temperature (°C)	0.64 (0.56, 0.72)	<0.001
Norepinephrine, yes vs. no	2.54 (2.10, 3.08)	<0.001
Lactate (mmol/L)	1.15 (1.12, 1.19)	<0.001
WBC_max (K/uL)	1.00 (1.00, 1.01)	<0.001
Potassium_max (K/uL)	1.12 (1.03, 1.22)	0.006
INR_max		
1.2–1.4 vs. ≦1.2	0.80 (0.57, 1.11)	0.176
>1.4 vs. ≦1.2	1.47 (1.12, 1.96)	0.006
PT_max (s)		
15-18 vs. ≦15	0.94 (0.75, 1.20)	0.641
18-21 vs. ≦15	1.47 (1.12, 1.94)	0.006
>21 vs. ≦15	2.41 (1.89, 3.07)	<0.001
RDW_max (%)	1.23 (1.19, 1.28)	<0.001
MCV_min (fL)	1.05 (1.04, 1.06)	<0.001
Creatinine_max (μmol/L)		
110–170 vs. <110	1.43 (1.14, 1.80)	0.002
171–299 vs. <110	1.89 (1.49, 2.41)	<0.001
300–440 vs. <110	2.94 (2.09, 4.14)	<0.001
>440 vs. <110	2.10 (1.52, 2.89)	<0.001
Lowest platelet level (K/uL)	0.99 (0.98, 0.99)	<0.001

*PT, Prothrombin Time; INR, International Normalized Ratio; RDW, Red Blood Cell Distribution Widths; MCV, Mean Corpuscular Volume; MAP, Mean arterial pressure; OR, odds rate; CI, confidence interval*.

**Figure 1:**
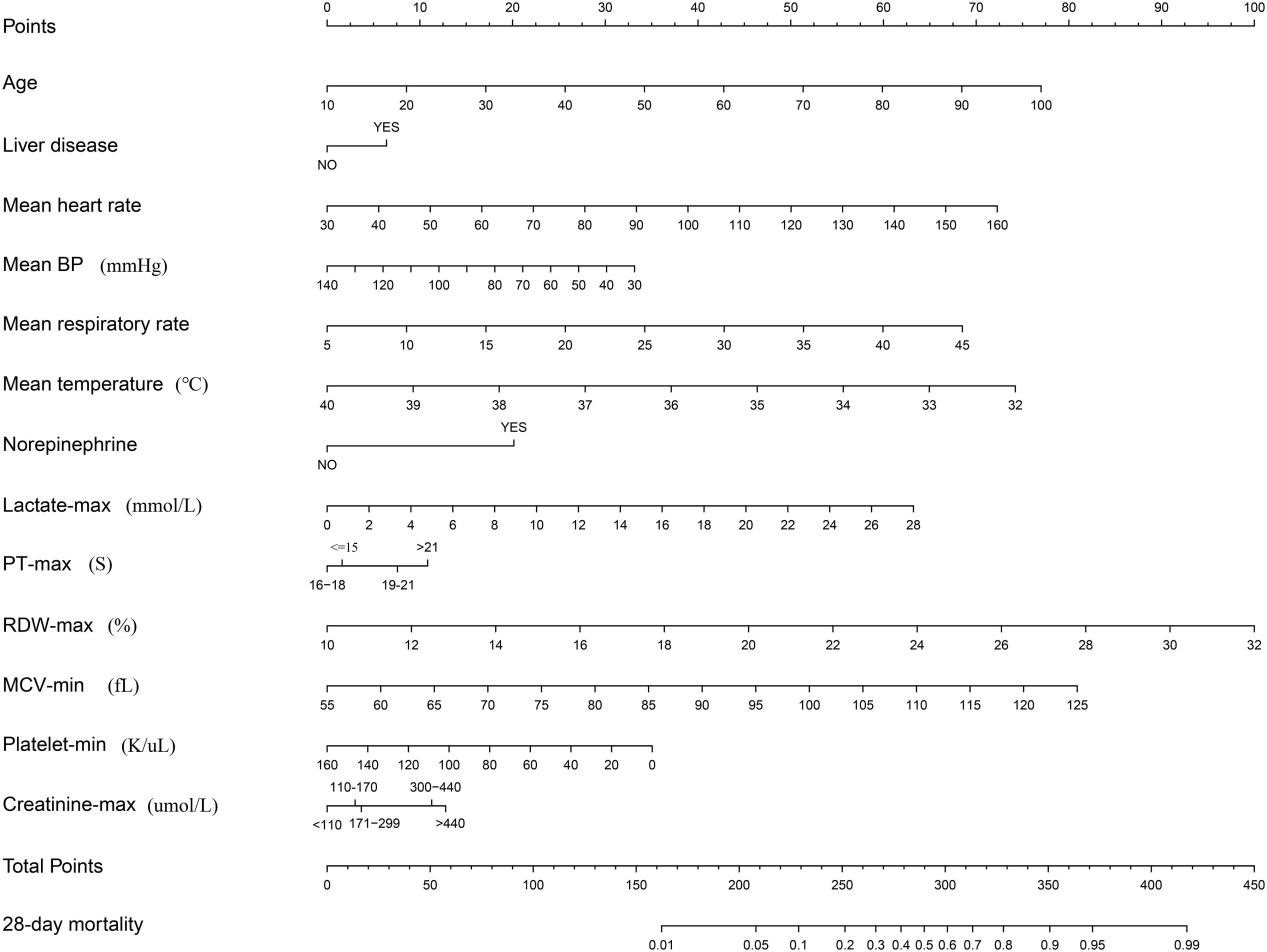
Nomogram to predict the risk of 28-day mortality of patients with SIC. When using it, drawing a vertical line from each variable to the points axis for the score, then the points for all the parameters were added, finally, a line from the total points axis was drawn to correspond the risk of 28-day mortality at the bottom.

Subsequently, a multivariate logistic regression was performed using variables with *p* < 0.05 in the univariate logistic analysis or those that had clinical significance or these categorical variables in which a set of meaningful values existed. However, the infection site and PH-min were omitted from the model, considering that it was difficult to determine the source of infection in the early stage of ICU admission and the PH value was affected by a variety of factors. Finally, we selected a total of 13 variables based on the AIC. The risk factors independently associated with the 28-day mortality of SIC identified by the multivariable analysis are presented in Table 3. Regarding collinearity, the VIF of all continuous variables in Table 3 was <2, indicating that no collinearity existed in the regression analysis. Next, a model integrating age, combined with liver disease, mean arterial pressure (MAP), mean heart rate, mean respiratory rate, mean temperature, the administration of norepinephrine, lactate-max, PT-max, RDW-max, MCV-min, creatinine-max and lowest platelet level was established using the training set. On the basis of this model, a nomogram was plotted to predict the probability of the 28-day mortality of the SIC patients (Figure 1).

**Table 3:** Factors independently associated with 28-day mortality of patients with SIC by multivariate logistic regression analysis in training cohort.

**Variables**	**β^a^**	**OR (95% CI)**	***p*-values**
Age, y	0.03	1.03 (1.02, 1.04)	<0.001
Liver-disease, yes vs. no	0.23	1.26 (0.96, 1.65)	0.091
MAP (mmHg)	−0.01	0.99 (0.98, 1.00)	0.033
Mean heart rate (min^−1^)	0.02	1.02 (1.01, 1.03)	<0.001
Mean respiratory rate (min^−1^)	0.06	1.06 (1.04, 1.10)	<0.001
Mean temperature (°C)	−0.33	0.72 (0.62, 0.83)	<0.001
Norepinephrine, yes vs. no	0.73	2.07 (1.66, 2.57)	<0.001
Lactate (mmol/L)	0.08	1.11 (1.05, 1.12)	<0.001
PT_max (s)			
15-18 vs. ≦15	−0.27	0.76 (0.58, 0.99)	0.045
18-21 vs. ≦15	−0.21	0.81 (0.58, 1.11)	0.189
>21 vs. ≦15	0.11	1.12 (083, 1.52)	0.440
RDW_max (%)	0.16	1.18 (1.13, 1.23)	<0.001
MCV_min (fL)	0.04	1.04 (1.03, 1.06)	<0.001
Lowest platelet level (K/uL)	−0.01	0.99 (0.98, 0.99)	<0.001
Creatine_max (μmol/L)			
110–170 vs. <110	0.13	1.14 (0.88, 1.48)	0.312
171–299 vs. <110	0.11	1.12 (0.84, 1.48)	0.453
300–440 vs. <110	0.46	1.59 (1.07, 2.35)	0.022
>440 vs. <110	0.41	1.50 (1.04, 2.16)	0.030

*PT, Prothrombin Time; RDW, Red Blood Cell Distribution Widths; MCV, Mean Corpuscular Volume; MAP, Mean arterial pressure*.

a *Unstandardized β coefficients were calculated from the multivariate logistic regression model*.

*OR, odds rate; CI, confidence interval*.

### Validation of the Prediction Nomogram

**Figure 2:**
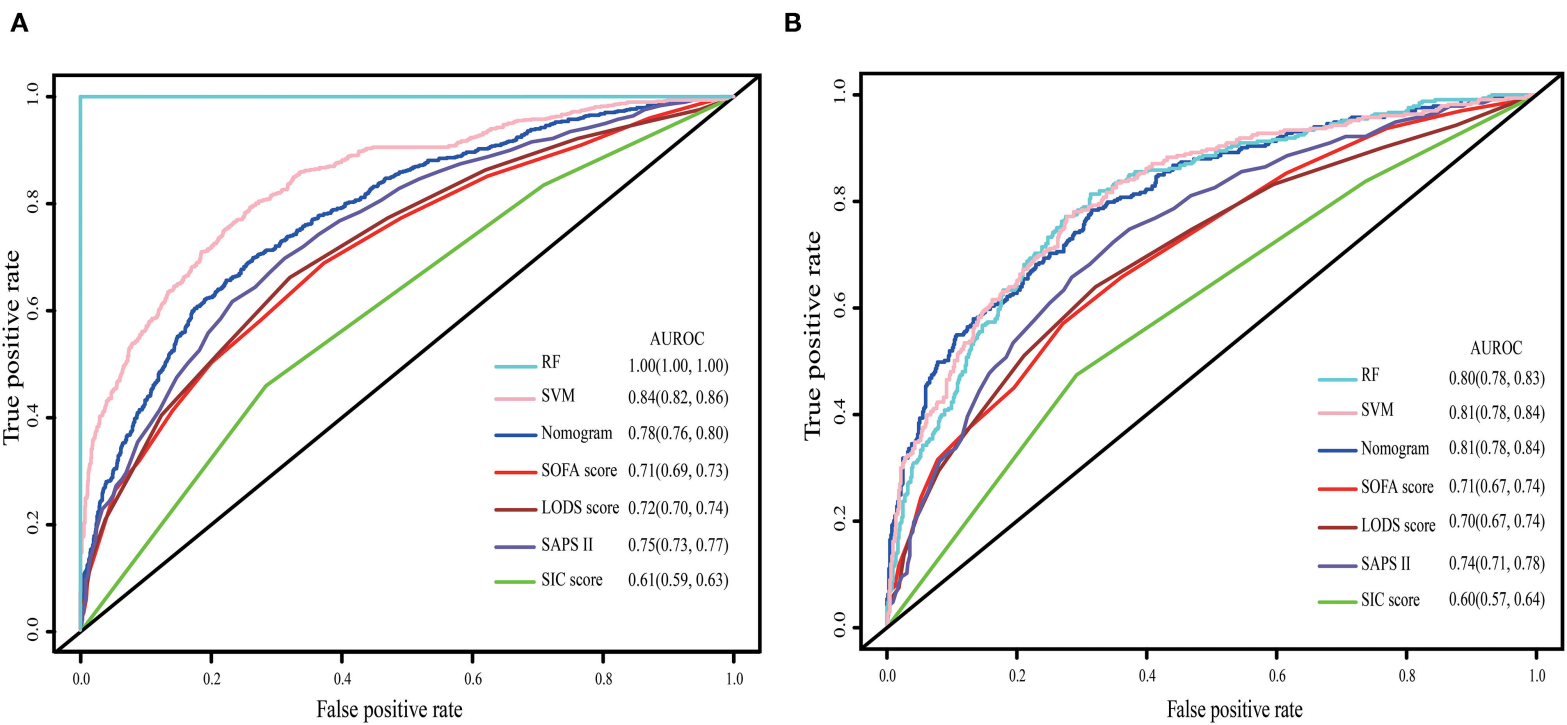
The ROC curve of the nomogram, RF model, SVM model, SOFA, LODS, SAPS II and SIC. **(A)** Training set; **(B)** Validation set. The variables entered in nomogram, RF model and SVM model are the same.

The nomogram demonstrated good accuracy for predicting the 28-day mortality of SIC patients, with an unadjusted C-index of 0.78 (95% CI: 0.76, 0.80). In the validation set, the nomogram displayed an unadjusted C-index of 0.81 (95% CI: 0.78, 0.84). The nomogram when compared with the SOFA, LODS, SAPS II, and SIC scores displayed an area under the receiver operating characteristic (AUROC) that was significantly higher in both sets. Furthermore, the RF and SVM models showed an excellent ability to distinguish the SIC patients who died during the 28 days since admission in the training cohort, but it declined sharply in the validation cohort (Figure 2).

**Figure 3:**
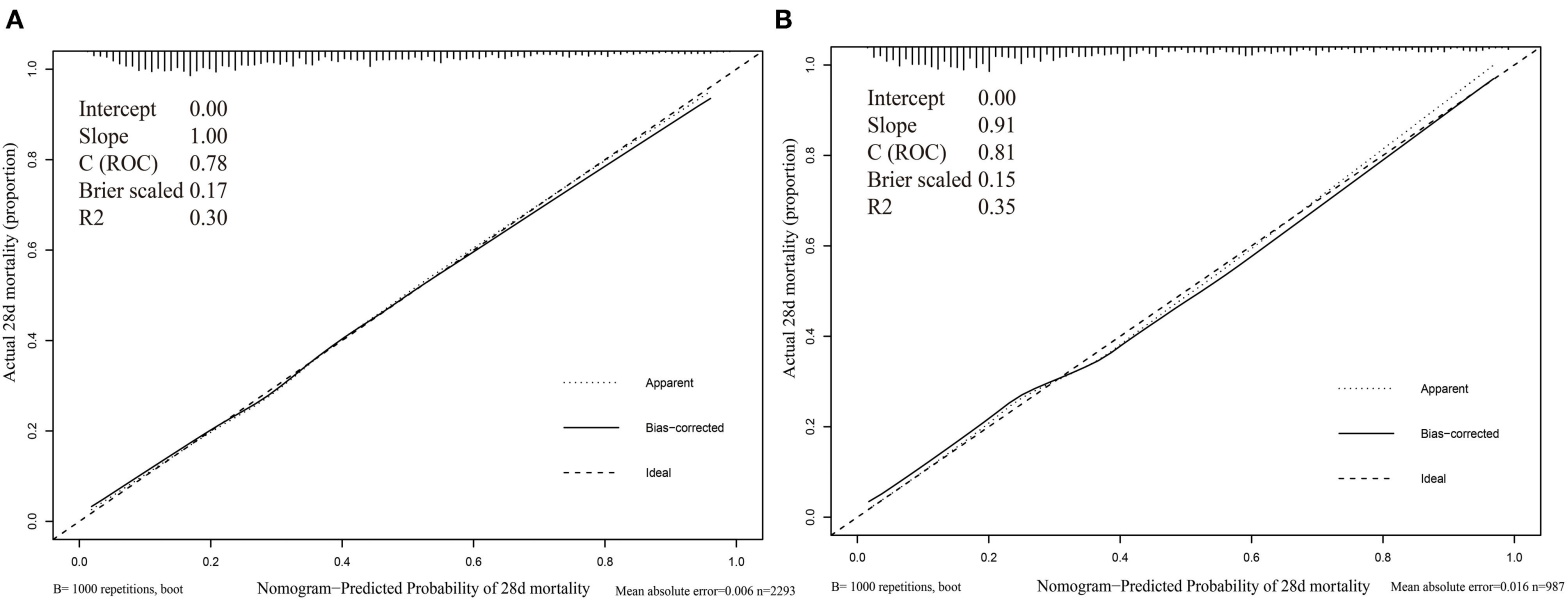
Calibration curves of nomogram. **(A)** Training set; **(B)** Validation set.

The calibration curve was described using the bootstrap method for both, the training and validation sets (Figure 3). The apparent line and a bias-corrected line only slightly deviated from the ideal line, indicating a good agreement between the prediction and reality. The Brier score of the nomogram was 0.17 and 0.15 in the training and validation sets, respectively. The IDI and NRI indices of the nomogram were also significantly higher than those of the SOFA, LODS, SAPS II, and SIC scores in both sets, as shown in Table 4, which indicated that this nomogram had a better prediction probability in 28-day mortality prediction.

**Table 4:** Comparison of models in predicting the 28-day mortality of patients with SIC.

**Predictive model**		**AUROC**	***P*-value**	**IDI**	***P*-value**	**NRI**	***P*-value**
**Training set**	Nomogram	0.78 (0.76, 0.80)					
	SOFA	0.71 (0.69, 0.73)	<0.001	0.09 (0.007, 0.11)	<0.001	0.30 (0.20, 0.47)	<0.001
	LODS	0.72 (0.70, 0.74)	<0.001	0.08 (0.06, 0.10)	<0.001	0.17 (0.06, 0.29)	<0.001
	SAPS II	0.75 (0.73, 0.77)	0.01	0.05 (0.03, 0.07)	<0.001	0.12 (0.08, 0.22)	<0.001
	SIC score	0.61 (0.59, 0.63)	<0.001	0.12 (0.08, 0.22)	<0.001	0.54 (0.39, 0.62)	<0.001
**Validation set**	Nomogram	0.81 (0.78, 0.84)					
	SOFA	0.71 (0.67, 0.74)	<0.001	0.15 (0.12, 0.18)	<0.001	0.40 (0.24, 0.53)	<0.001
	LODS	0.70 (0.67, 0.74)	<0.001	0.16 (0.13, 0.19)	<0.001	0.34 (0.23, 0.46)	<0.001
	SAPS II	0.74 (0.71, 0.78)	<0.001	0.12 (0.09, 0.15)	<0.001	0.23 (0.17, 0.31)	<0.001
	SIC score	0.60 (0.57, 0.64)	<0.001	0.25 (0.22, 0.28)	<0.001	0.58 (0.42, 0.65)	<0.001

*The P-value was calculated by comparing the results of nomogram with SOFA or LODS, SAPS II, and SIC score*.

*AUROC, area under the receiver operating characteristic curve; IDI, integrated discrimination improvement; NRI, net reclassification improvement; SOFA, Sequential Organ Failure Assessment; LODS, Logistic Organ Dysfunction System; SAPS II, Simplified acute physiology II*.

### Clinical Use of the Nomogram

**Figure 4:**
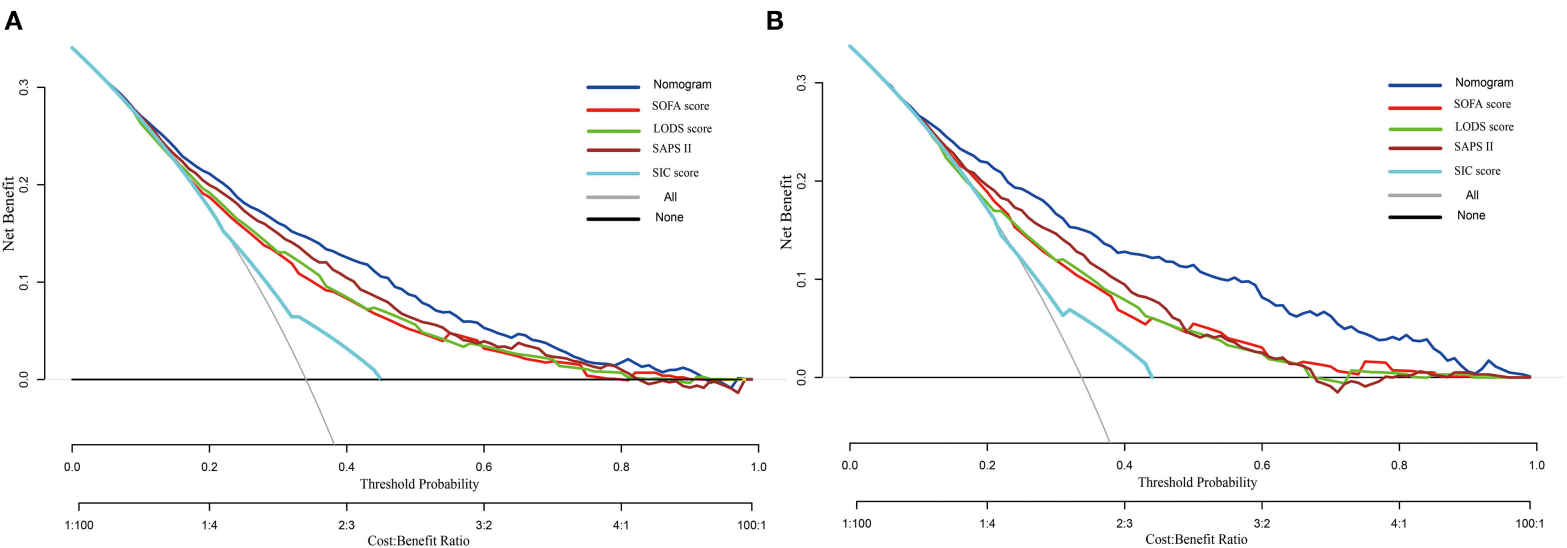
Decision curve analysis of the nomogram, SOFA, LODS, SAPS II and SIC. **(A)** Training set; **(B)** Validation set.

The DCA curve was plotted to perform a clinical application of this nomogram, and compared with other clinical severity scoring systems. In the training set, clinical intervention guided by this nomogram provided a greater net benefit when the threshold probability was within 0.1 and 0.9 (Figure 4A). In the validation set, the analysis indicated that when the threshold probability was >0.15, using this nomogram to predict the 28-day mortality of SIC patients could provide a greater net benefit than the SOFA, LODS, and SAPS II (Figure 4B). However, we found that the SIC score performed the worst. When the threshold probability was >0.45, the DCA curve of the SIC score overlapped with the horizontal line.

On the basis of the DCA, the clinical impact curve for this nomogram is presented (Supplementary Figure 4). In both sets, the red solid curve (number of high-risk individuals) represented the number of patients classified as high risk by this nomogram under each risk threshold of 1,000 patients, and the blue dashed curve (number of high-risk individuals with outcome) showed the number of true positive patients under each risk threshold.

### Risk of 28-day Mortality Based on the Nomogram Scores

The results showed that this nomogram is a good predictive model, with high sensitivity, specificity, positive predictive value, and negative predictive value in recognizing whether the patients survived or were deceased after 28 days since ICU admission, with 0.70 (95% CI: 0.67, 0.73), 0.74 (95% CI: 0.71, 0.76), 0.58 (95% CI: 0.55, 0.62) and 0.83 (95% CI: 0.80, 0.84) in the training set, and 0.78 (95% CI: 0.74, 0.83), 0.69 (95% CI: 0.65, 0.72), 0.56 (95% CI: 0.52, 0.63), and 0.86 (95% CI: 0.83, 0.88) in the validation set, respectively (Supplementary Table 2).

## Discussion

In this retrospective cohort study of a large open-source database, univariate and multivariate logistic regression analyses were successively applied to identify the independent risk factor associated with the 28-day mortality of SIC patients in the ICU. Finally, a total of 13 clinical variables were recognized and incorporated into a best-fit model, that is, the age, mean heart rate, MAP, mean respiratory rate, mean temperature, lactate-max, PT-max, RDW-max, MCV-min, creatinine-max, lowest level of platelet count, the administration of norepinephrine and combined with liver disease.

The results showed a SIC incidence of 34.8% and a 28-day mortality of 34.0%. These rates were higher than in previous reports (6, 9). Only sepsis patients admitted to the ICU were included in the present study; therefore, population diversity could explain these differences. Most SIC patients were male and commonly found in the medical ICU. Moreover, patients who had SIC displayed a significantly reduction in their short-term survival by the Kaplan–Meier's survival analysis and a prolonged hospitalization time compared with non-SIC patients. These findings were similar to those of Lyons et al. (18). Interestingly, some related comorbidities, including diabetes and COPD, were less prevalent in the SIC cohort. This tendency was also displayed in another study (18).

Among the thirteen included variables, the RDW was a major factor. Indeed, it was the strongest predictor for 28-day mortality in terms of relative contribution. The RDW is a routine parameter in reflecting the heterogeneity of erythrocyte cell size and discriminating anemic types (19). Numerous studies have recently revealed a significant association between the RDW value and increased mortality in sepsis patients (20, 21). A large cohort study that included 11,691 sepsis patients demonstrated that the initial RDW within the first 24 h of admission was an independent risk factor for the 28-day mortality. For every one unit increase in the RDW value, the 28-day mortality increased by 6.86% (20). During the first 72 h of hospitalization, the extent of the rise in the RDW value was also associated with a poorer prognosis of sepsis patients or septic shock patients (21). Although the underlying mechanism was unclear, several possible reasons could explain the correlation between the RDW and sepsis patient mortality. The systemic inflammation response can impact the status of hematopoietic organs. In fluorodeoxyglucose positron emission tomography (FDG-PET) scanning, an association between the RDW and splenic and lumbar bone marrow activation was revealed (22). Furthermore, previous research proved that inflammation could suppress erythrocyte maturation and accelerate reticulocyte transfer into the peripheral circulation (23). Another explanation may be related to high oxidative stress. The excessive expression of reactive oxygen species induced severe cellular dysfunctions or even MODS in sepsis patients (24).

Several other parameters in the nomogram were associated with sepsis or coagulation abnormalities. Epidemiological data demonstrated that age is an independent risk factor for thrombosis and is associated with the 90-day and 1-year mortalities in sepsis patients (25–27). During sepsis, the incidence of liver dysfunction approaches 34–46% (28). When sepsis patients also had a liver disease, including cirrhosis and tumor, the risks for MODS and mortality were significantly higher than in patients without liver diseases (29). Vital signs were widely used to develop the prediction model of sepsis (30, 31) and were also included in the nomogram. Furthermore, SIC was normally characterized by reduced platelets and prolonged PT or INR. Notably, a decreased mortality rate of SIC patients was found in the present study when the PT values ranged from 16 to 18s. We supposed that a mildly prolonged PT might be more likely to gain the attention of the physician than a normal PT, which in turn would lead to earlier intervention. Alteration of the lactic levels reflects the situation of the microcirculatory perfusion. When lactic levels were >2.5 mmol/L, the probability of mortality increased with increasing lactic concentration, and this correlation was independent of vasopressor administration (32, 33).

Currently, no specialized prediction models for the assessment of the 28-day mortality risk in SIC patients are available. As defined in the Surviving Sepsis Campaign 2016 guideline, sepsis is induced by infections and eventually leads to systemic multiple organ dysfunction. Therefore, several scoring systems applied to evaluate organ functional status were useful in predicting the prognosis of sepsis patients. The SOFA and LODS were widely applied in the ICU, and may be more appropriate to reflect the acute changes in organ function of sepsis patients (34). However, the effectiveness of these scoring systems in predicting the 28-day mortality risk of SIC patients remained unknown. Therefore, we compared the predictive ability of the proposed nomogram with some common clinical rating scales, including the SOFA, LODS, SAPS II and SIC score, based on the AUROC. We found that the nomogram performed best. Furthermore, the DCA curve and IDI and NRI indices also supported this conclusion. Additionally, the nomogram could effectively discriminate the real positive patients with a high risk for 28-day mortality in both the training and validation sets. In the present study, we attempted to develop other machine-learning models, including RF and SVM, to improve the accuracy of the prediction. However, the AUROC of these models decreased dramatically in the process of validation, which indicated poor generalization ability. On the basis of predictive power and clinical interpretability, we chose multivariate logistic regression as the final model to construct the proposed nomogram. However, we are currently developing an XGBoost model using a new external database.

The nomogram developed here performed well in the discrimination of 28-day mortality risk, as reflected by a high C-index of 0.81 and an acceptable calibration. When obtaining a nomogram, physicians only need to calculate the scores corresponding to each indicator based on the first row, and then add up each point to obtain a final total points value. Finally, the 28-day mortality can be determined based on the final row. In the calculation process, vital signs and the laboratory test values of the SIC patients during the first 24 h since ICU admission are necessary.

The present study also had several limitations. First, according to the sepsis 3.0 criterion, infection and suspected infection diagnosing requires an exact time of the sampling culture and antibiotic use. These were difficult to obtain from the MIMIC III database. Therefore, we referred to the Angus criterion to extract the infectious patients (35). Second, in the PT were inherent defects reflecting the pro-coagulant and anti-coagulant processes (36, 37). Some new coagulation markers and examinations, including thrombin-antithrombin-III complex, plasmin-α2-antiplasmin complex and thromboelastography, are becoming useful tools in coagulopathy diagnosis (38, 39). Combining these parameters with the current optimization model may further optimize the capacity for 28-day mortality prediction in SIC patients; however, they were not recorded in the MIMIC III database. Third, nomogram as a visualization tool, could make the analyses more intuitive and convenient, but it has been used for years. In addition to nomogram, clinical scoring scale and web-based risk calculators were commonly used. For some models that are harder to explain, such as integrated tree model and neural network model, SHAP algorithm may be useful. In recent years, increasing efforts have been put into improving the interpretability of black-box artificial intelligence and designing more interpretable models for clinical prediction (40, 41). This will be our future direction.

In conclusion, on the basis of logistic regression analysis, a nomogram including 13 conventional clinical variables was conducted. This model provided an optimal prediction of the 28-day mortality risk in SIC patients and through the internal validation. Using this model, the 28-day mortality risk of an individual SIC patient can be determined, which can lead to an improved prognostic assessment. However, external validation is required for further generalizability improvement of this nomogram.

## Data Availability Statement

All available data were obtained from MIMIC-III database, further inquiries can be directed to the corresponding author/s.

## Ethics Statement

Ethical review and approval was not required for the study on human participants in accordance with the local legislation and institutional requirements. Written informed consent for participation was not required for this study in accordance with the national legislation and the institutional requirements.

## Author Contributions

MY and JZ: concept. ZL and JZ: methodology and writing of the manuscript and contributed equally. JH and JW: data processing. YL and WX: software. MY and TH: review and editing. All authors contributed to the article and approved the submitted version.

## Conflict of Interest

The authors declare that the research was conducted in the absence of any commercial or financial relationships that could be construed as a potential conflict of interest.

## Supplementary Material

Supplementary Figure 1 Flowchart of data extraction and study design.

Supplementary Figure 2 K-M curves estimated the 7-day **(A)** and 28-day **(B)** survival probability of SIC and non-SIC patients. The log-rank results showed that the 7-day and 28-day survival of SIC patients was significantly lower than that of non-SIC patients.

Supplementary Figure 3 After PSM processed, K-M curves estimated the 7-day **(A)** and 28-day **(B)** survival probability of SIC and non-SIC patients. The log-rank results showed that the 7-day and 28-day survival of SIC patients was significantly lower than that of non-SIC patients.

Supplementary Figure 4 The clinical impact curve of the nomogram, in which red solid curve indicates the number of people who are classified as high risk by the nomogram at each threshold probability; the blue dashed curve showed the number of true positive patients under each risk threshold. **(A)** Training set; **(B)** Validation set.

Supplementary Table 1 The characteristics of SIC patients in the training set and validation set.

Supplementary Table 2 Accuracy of the nomogram for predicting the risk of 28-day mortality in SIC patients.

## References

[1] Levi M van der Poll T. Coagulation and sepsis. Thromb Res. (2017) 149:38–44. 10.1016/j.thromres.2016.11.007 27886531

[2] Fleischmann-Struzek C Mellhammar L Rose N Cassini A Rudd KE Schlattmann P. Incidence and mortality of hospital- and ICU-treated sepsis: results from an updated and expanded systematic review and meta-analysis. Intensive Care Med. (2020) 46:1552–62. 10.1007/s00134-020-06151-x 32572531 PMC7381468

[3] Simmons J Pittet JF. The coagulopathy of acute sepsis. Curr Opin Anaesthesiol. (2015) 28:227–36. 10.1097/ACO.0000000000000163 25590467 PMC4407200

[4] Lipinska-Gediga M. Coagulopathy in sepsis - a new look at an old problem. Anaesthesiol Intensive Ther. (2016) 48:352–9. 10.5603/AIT.a2016.0051 27824218

[5] Vincent JL Francois B Zabolotskikh I Daga MK Lascarrou JB Kirov MY. Effect of a recombinant human soluble thrombomodulin on mortality in patients with sepsis-associated coagulopathy: the SCARLET randomized clinical trial. JAMA. (2019) 321:1993–2002. 10.1001/jama.2019.5358 31104069 PMC6547077

[6] Saito S Uchino S Hayakawa M Yamakawa K Kudo D Iizuka Y. Epidemiology of disseminated intravascular coagulation in sepsis and validation of scoring systems. J Crit Care. (2019) 50:23–30. 10.1016/j.jcrc.2018.11.009 30471557

[7] Tiru B DiNino EK Orenstein A Mailloux PT Pesaturo A Gupta A. The economic and humanistic burden of severe sepsis. Pharmacoeconomics. (2015) 33:925–37. 10.1007/s40273-015-0282-y 25935211

[8] Jhang WK Park SJ. Evaluation of sepsis-induced coagulopathy in critically ill pediatric patients with septic shock. Thromb Haemost. (2020). 10.1055/s-0040-1718736. [Epub ahead of print]. 33124023

[9] Iba T Nisio MD Levy JH Kitamura N Thachil J. New criteria for sepsis-induced coagulopathy (SIC) following the revised sepsis definition: a retrospective analysis of a nationwide survey. BMJ Open. (2017) 7:e017046. 10.1136/bmjopen-2017-017046 28963294 PMC5623518

[10] Iba T Arakawa M Di Nisio M Gando S Anan H Sato K. Newly proposed sepsis-induced coagulopathy precedes international society on thrombosis and haemostasis overt-disseminated intravascular coagulation and predicts high mortality. J Intensive Care Med. (2020) 35:643–9. 10.1177/0885066618773679 29720054

[11] Ding R Wang Z Lin Y Liu B Zhang Z Ma X. Comparison of a new criteria for sepsis-induced coagulopathy and International Society on Thrombosis and Haemostasis disseminated intravascular coagulation score in critically ill patients with sepsis 3.0: a retrospective study. Blood Coagul Fibrinolysis. (2018) 29:551–8. 10.1097/MBC.0000000000000755 30015646 PMC6133197

[12] Li X Fan Y Dong Y Cheng Y Zhou J Wang Z. Development and validation of nomograms predicting the overall and the cancer-specific survival in endometrial cancer patients. Front Med. (2020) 7:614629. 10.21203/rs.3.rs-68463/v1 33425959 PMC7785774

[13] Xun Y Chen M Liang P Tripathi P Deng H Zhou Z. A novel clinical-radiomics model pre-operatively predicted the stone-free rate of flexible ureteroscopy strategy in kidney stone patients. Front Med. (2020) 7:576925. 10.3389/fmed.2020.576925 33178719 PMC7593485

[14] Ge H Jiang Y Jin Q Wan L Qian X Zhang Z. Nomogram for the prediction of postoperative hypoxemia in patients with acute aortic dissection. BMC Anesthesiol. (2018) 18:146. 10.1186/s12871-018-0612-7 30342471 PMC6195757

[15] Johnson AE Pollard TJ Shen L Lehman LW Feng M Ghassemi M. MIMIC-III, a freely accessible critical care database. Sci Data. (2016) 3:160035. 10.1038/sdata.2016.35 27219127 PMC4878278

[16] Collins GS Reitsma JB Altman DG Moons KG. Transparent Reporting of a multivariable prediction model for Individual Prognosis Or Diagnosis (TRIPOD). Ann Intern Med. (2015) 162:735–6. 10.7326/L15-5093-2 25984857

[17] Singer M Deutschman CS Seymour CW Shankar-Hari M Annane D Bauer M. The third international consensus definitions for sepsis and septic shock (Sepsis-3). JAMA. (2016) 315:801–10. 10.1001/jama.2016.0287 26903338 PMC4968574

[18] Lyons PG Micek ST Hampton N Kollef MH. Sepsis-associated coagulopathy severity predicts hospital mortality. Crit Care Med. (2018) 46:736–42. 10.1097/CCM.0000000000002997 29373360

[19] Piriyakhuntorn P Tantiworawit A Rattanathammethee T Chai-Adisaksopha C Rattarittamrong E Norasetthada L. The role of red cell distribution width in the differential diagnosis of iron deficiency anemia and non-transfusiondependent thalassemia patients. Hematol Rep. (2018) 10:7605. 10.4081/hr.2018.7605 30283620 PMC6151350

[20] Huidong L Bo X. Evaluation of the influence of red blood cell distribution width on the prognosis of patients with sepsis based on data mining. J Clin Emerg. (2019) 20:263–7. 10.13201/j.issn.1009-5918.2019.04.002

[21] Kim CH Park JT Kim EJ Han JH Han JS Choi JY. An increase in red blood cell distribution width from baseline predicts mortality in patients with severe sepsis or septic shock. Crit Care. (2013) 17:R282. 10.1186/cc13145 24321201 PMC4056357

[22] Van Koeverden ID den Ruijter HM Scholtes VPW G EH Lam M Haitjema S Buijsrogge MP. A single preoperative blood test predicts postoperative sepsis and pneumonia after coronary bypass or open aneurysm surgery. Eur J Clin Invest. (2019) 49:e13055. 10.1111/eci.13055 30475403

[23] Straat M van Bruggen R de Korte D Juffermans NP. Red blood cell clearance in inflammation. Transfus Med Hemother. (2012) 39:353–61. 10.1159/000342229 23801928 PMC3678279

[24] Kolls JK. Oxidative stress in sepsis: a redox redux. J Clin Invest. (2006) 116:860–3. 10.1172/JCI28111 16585954 PMC1421363

[25] Mahé I Caulin C Bergmann JF. Age, an independent risk factor for thrombosis. Epidemiologic data. Presse Med. (2005) 34:878–86. 10.1016/S0755-4982(05)84068-0 16097213

[26] Xie JF Wang HL Kang Y Zhou LX Liu ZM Qin BY. The epidemiology of sepsis in Chinese ICUs: a national cross-sectional survey. Crit Care Med. (2020) 48:e209–19. 10.1097/CCM.0000000000004155 31804299

[27] He XL Liao XL Xie ZC Han L Yang XL Kang Y. Pulmonary infection is an independent risk factor for long-term mortality and quality of life for sepsis patients. Biomed Res Int. (2016) 2016:4213712. 10.1155/2016/4213712 28050557 PMC5165149

[28] Brun-Buisson C Meshaka P Pinton P Vallet B EPISEPSIS Study Group. EPISEPSIS: a reappraisal of the epidemiology and outcome of severe sepsis in French intensive care units. Intensive Care Med. (2004) 30:580–8. 10.1007/s00134-003-2121-4 14997295

[29] Yan J Li S Li S. The role of the liver in sepsis. Int Rev Immunol. (2014) 33:498–510. 10.3109/08830185.2014.889129 24611785 PMC4160418

[30] Faisal M Scally A Richardson D Beatson K Howes R Speed K. Development and external validation of an automated computer-aided risk score for predicting sepsis in emergency medical admissions using the patient's first electronically recorded vital signs and blood test results. Crit Care Med. (2018) 46:612–8. 10.1097/CCM.0000000000002967 29369828

[31] Churpek MM Snyder A Han X Sokol S Pettit N Howell MD. Quick sepsis-related organ failure assessment, systemic inflammatory response syndrome, and early warning scores for detecting clinical deterioration in infected patients outside the intensive care unit. Am J Respir Crit Care Med. (2017) 195:906–11. 10.1164/rccm.201604-0854OC 27649072 PMC5387705

[32] Thomas-Rueddel DO Poidinger B Weiss M Bach F Dey K Häberle H. Hyperlactatemia is an independent predictor of mortality and denotes distinct subtypes of severe sepsis and septic shock. J Crit Care. (2015) 30:439.e1-439.e4396. 10.1016/j.jcrc.2014.10.027 25466313

[33] Meiring C Dixit A Harris S MacCallum NS Brealey DA Watkinson PJ. Optimal intensive care outcome prediction over time using machine learning. PLoS ONE. (2018) 13:e0206862. 10.1371/journal.pone.0206862 30427913 PMC6241126

[34] Seymour CW Liu VX Iwashyna TJ Brunkhorst FM Rea TD Scherag A. Assessment of clinical criteria for sepsis: for the third international consensus definitions for sepsis and septic shock (Sepsis-3). JAMA. (2016) 315:762–74. 10.1001/jama.2016.0288 26903335 PMC5433435

[35] Angus DC Linde-Zwirble WT Lidicker J Clermont G Carcillo J Pinsky MR. Epidemiology of severe sepsis in the United States: analysis of incidence, outcome, and associated costs of care. Crit Care Med. (2001) 29:1303–10. 10.1097/00003246-200107000-00002 11445675

[36] Zeerleder S Hack CE Wuillemin WA. Disseminated intravascular coagulation in sepsis. Chest. (2005) 128:2864–75. 10.1378/chest.128.4.2864 16236964

[37] Scarlatescu E Juffermans NP Thachil J. The current status of viscoelastic testing in septic coagulopathy. Thromb Res. (2019) 183:146–52. 10.1016/j.thromres.2019.09.029 31678709

[38] Gall LS Davenport RA. Fibrinolysis and antifibrinolytic treatment in the trauma patient. Curr Opin Anaesthesiol. (2018) 31:227–33. 10.1097/ACO.0000000000000561 29324486

[39] Müller MC Meijers JC Vroom MB Juffermans NP. Utility of thromboelastography and/or thromboelastometry in adults with sepsis: a systematic review. Crit Care. (2014) 18:R30. 10.1186/cc13721 24512650 PMC4056353

[40] Xie F Chakraborty B Ong MEH Goldstein BA Liu N. AutoScore: a machine learning-based automatic clinical score generator and its application to mortality prediction using electronic health records. JMIR Med Inform. (2020) 8:e21798. 10.2196/21798 33084589 PMC7641783

[41] Zhang Z Navarese EP Zheng B Meng Q Liu N Ge H. Analytics with artificial intelligence to advance the treatment of acute respiratory distress syndrome. J Evid Based Med. (2020) 13:301–12. 10.1111/jebm.12418 33185950

