# Supplementary figures and images for: Development of a Nomogram to Predict 28-Day Mortality of Patients With Sepsis-Induced Coagulopathy: An Analysis of the MIMIC-III Database
Source: Front Med (Lausanne). 2021 Apr 6;8:661710. doi: 10.3389/fmed.2021.661710 (PMC8056034; doi:10.3389/fmed.2021.661710)

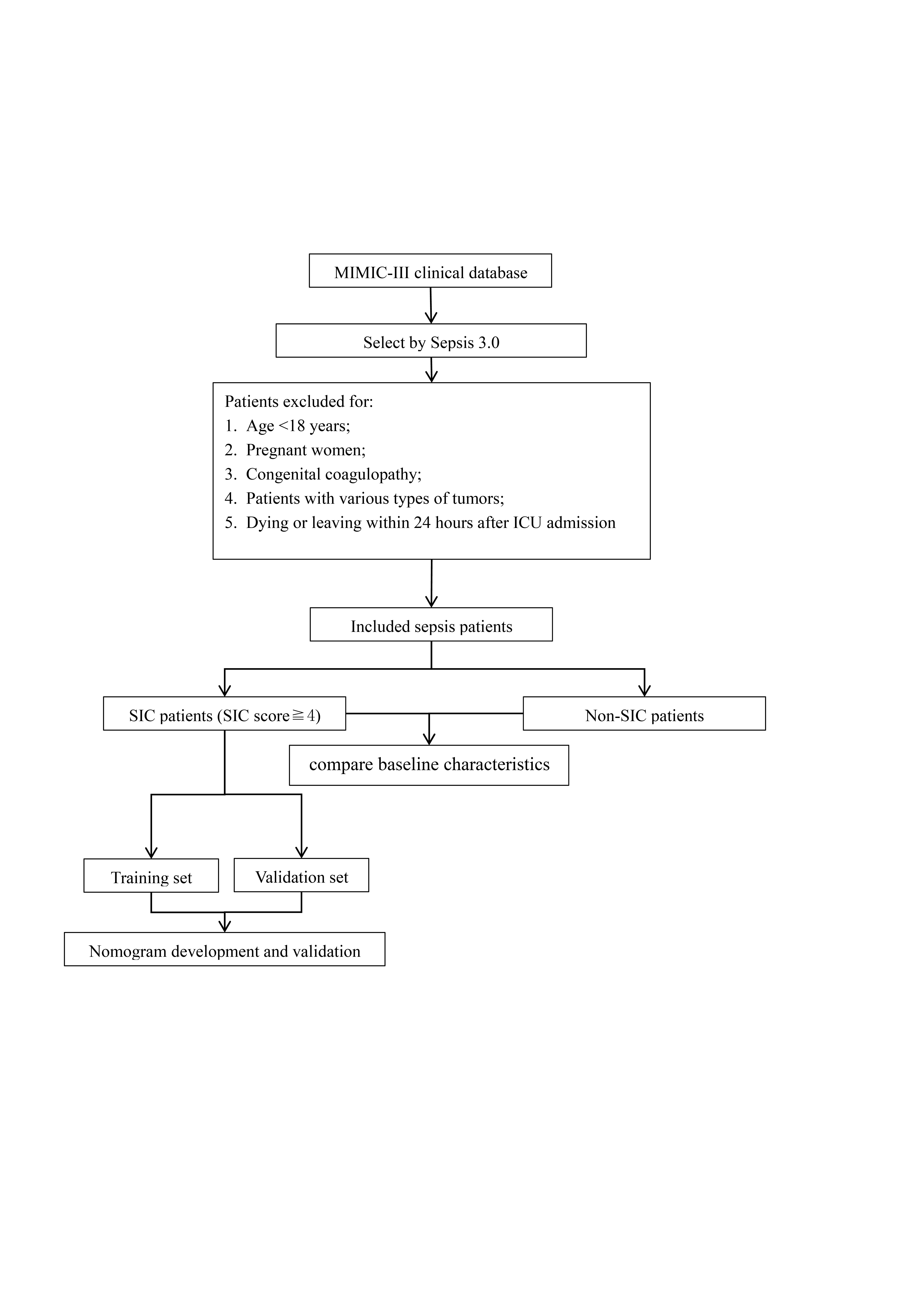

Supplement: Supplementary Figure 1 — Flowchart of data extraction and study design. [file Image_1.TIF]

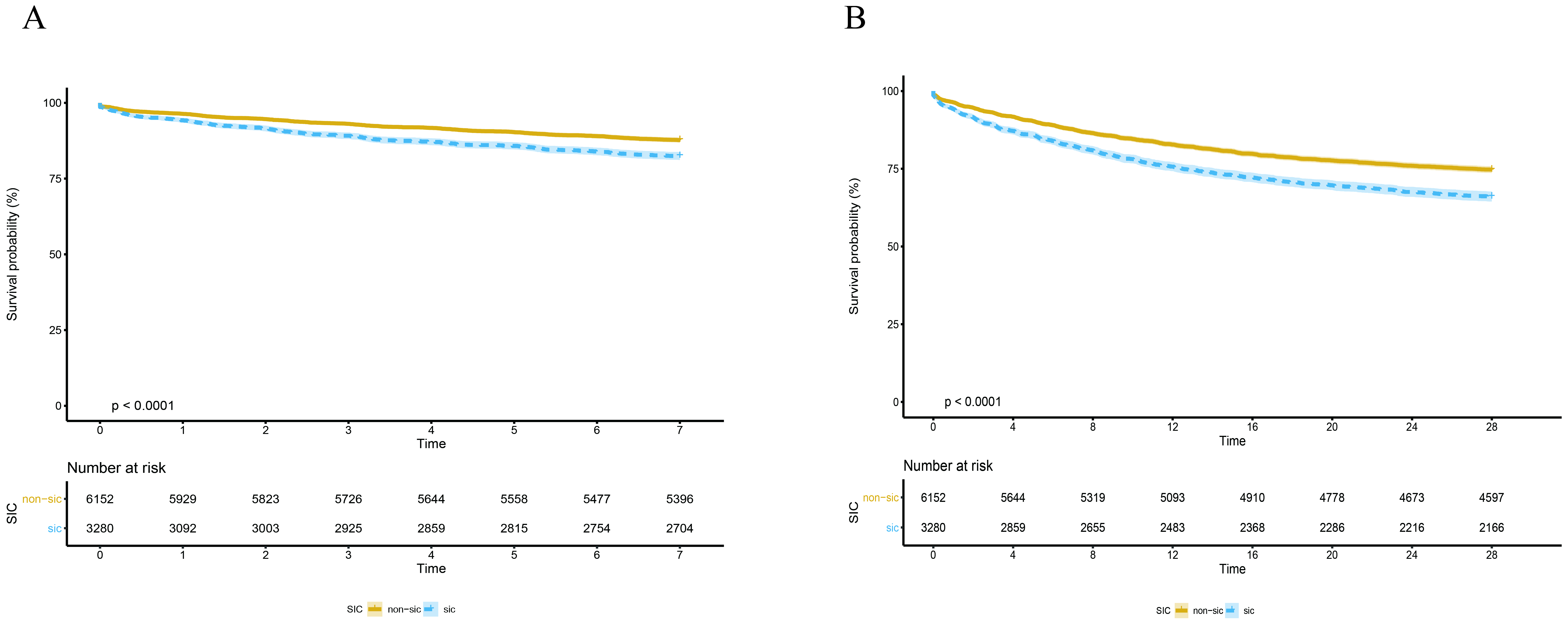

Supplement: Supplementary Figure 2 — K-M curves estimated the 7-day (A) and 28-day (B) survival probability of SIC and non-SIC patients. The log-rank results showed that the 7-day and 28-day survival of SIC patients was significantly lower than that of non-SIC patients. [file Image_2.TIF]

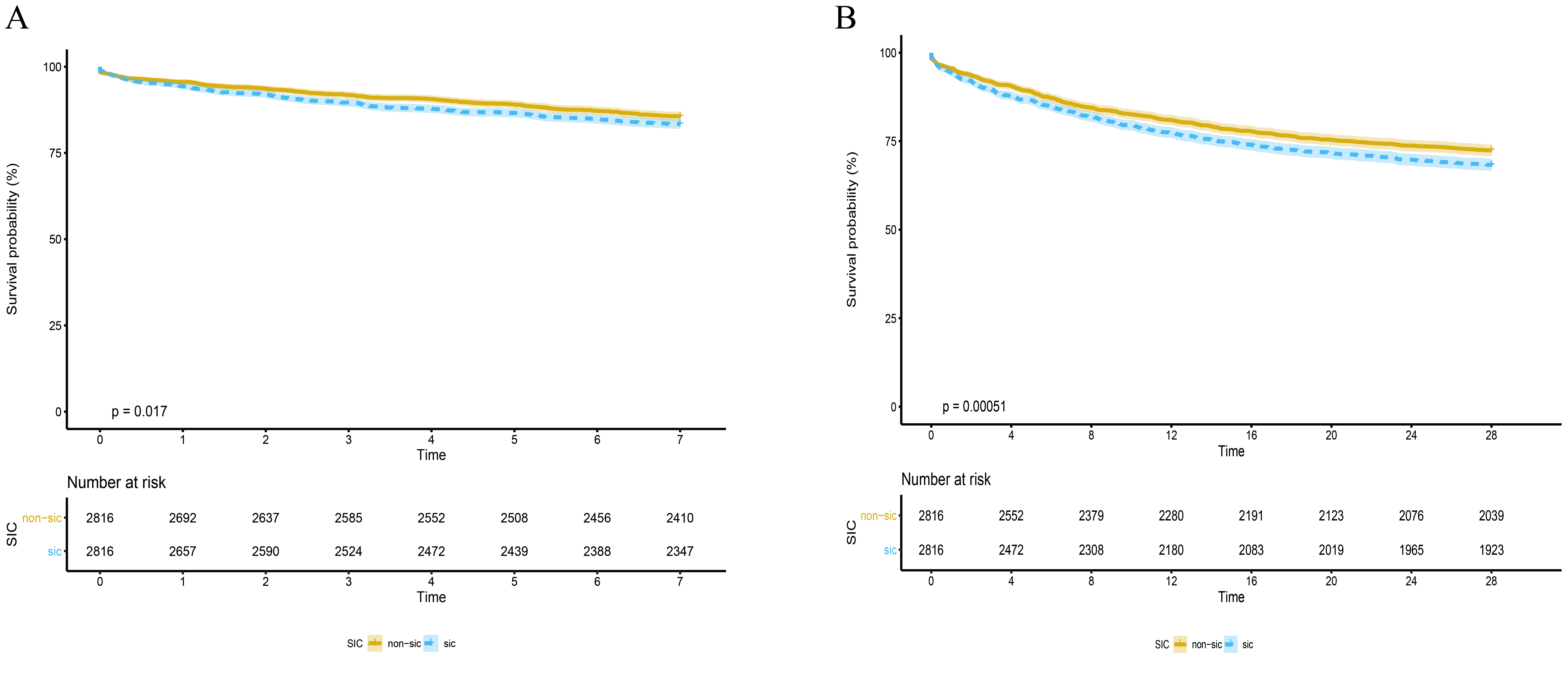

Supplement: Supplementary Figure 3 — After PSM processed, K-M curves estimated the 7-day (A) and 28-day (B) survival probability of SIC and non-SIC patients. The log-rank results showed that the 7-day and 28-day survival of SIC patients was significantly lower than that of non-SIC patients. [file Image_3.TIF]

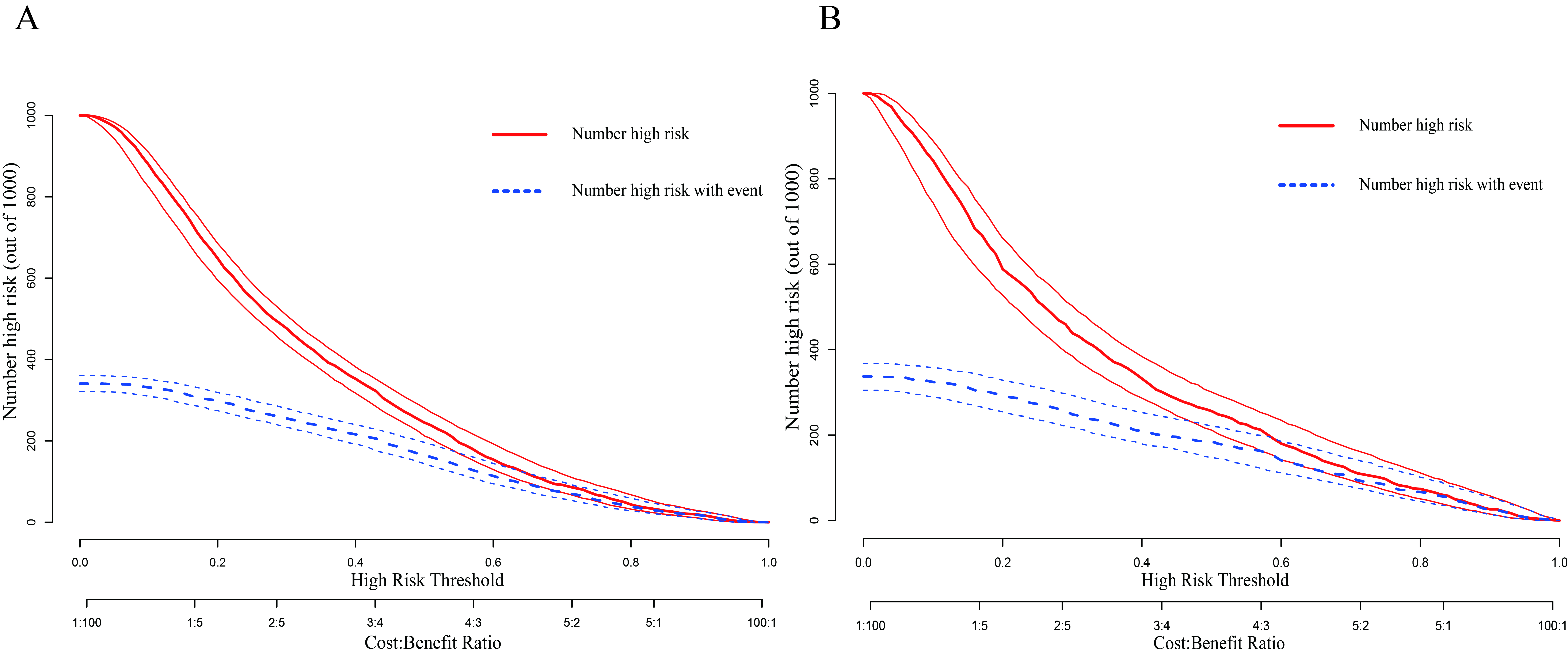

Supplement: Supplementary Figure 4 — The clinical impact curve of the nomogram, in which red solid curve indicates the number of people who are classified as high risk by the nomogram at each threshold probability; the blue dashed curve showed the number of true positive patients under each risk threshold. (A) Training set; (B) Validation set. [file Image_4.TIF]
