# Supplementary material for: Development of a Nomogram to Predict 28-Day Mortality of Patients With Sepsis-Induced Coagulopathy: An Analysis of the MIMIC-III Database
Source: Front Med (Lausanne). 2021 Apr 6;8:661710. doi: 10.3389/fmed.2021.661710 (PMC8056034; doi:10.3389/fmed.2021.661710)
Supplement: Supplementary Table 1 — The characteristics of SIC patients in the training set and validation set. [file Table_1.DOC]

Additional file Table 1 The characteristics of SIC patients in the training set and validation set

| **Variables** | **SIC patients (n = 3280)** | **Training set (n = 2293)** | **Validation set (n = 987)** | ***P*** |
| --- | --- | --- | --- | --- |
| Gender, n (%) |  |  |  | 0.999 |
| Male | 1959 (60) | 1369 (60) | 590 (60) |  |
| Female | 1321 (40) | 924 (40) | 397 (40) |  |
| Age, years | 66.93 (53.52, 79.08) | 67.00 (53.27, 79.04) | 66.77 (54.15, 79.16) | 0.869 |
| Weight (Kg) | 79.25 (66.48, 94.00) | 79.32 (67.00, 94.00) | 79.20 (65.80, 94.15) | 0.714 |
| **First care unit, n(%)** |  |  |  | 0.459 |
| CCU | 315 (10) | 221 (10) | 94 (10) |  |
| CSRU | 394 (12) | 280 (12) | 114 (12) |  |
| MICU | 1834 (56) | 1279 (56) | 555 (56) |  |
| SICU | 438 (13) | 316 (14) | 122 (12) |  |
| TSICU | 299 ( 9) | 197 ( 9) | 102 (10) |  |
| **Outcome** |  |  |  |  |
| ICU stay time, days | 4.21 (1.96, 10.04) | 4.25 (2.04, 9.88) | 4.08 (1.92, 10.10) | 0.53 |
| 7-day mortality, n(%) | 576 (18) | 401 (17) | 175 (18) | 0.907 |
| 28-day mortality, n(%) | 1114 (34) | 781 (34) | 333 (34) | 0.89 |
| Hospital mortality, n(%) | 1072 (33) | 745 (32) | 327 (33) | 0.75 |
| **Comorbidity, n(%)** |  |  |  |  |
| Hypertension, n(%) | 1040 (32) | 708 (31) | 332 (34) | 0.129 |
| COPD, n(%) | 70 ( 2) | 54 ( 2) | 16 ( 2) | 0.229 |
| Diabetes, n(%) | 870 (27) | 614 (27) | 256 (26) | 0.648 |
| MI, n(%) | 82 ( 2) | 58 ( 3) | 24 ( 2) | 0.966 |
| Cirrhosis, n(%) | 353 (11) | 240 (10) | 113 (11) | 0.441 |
| CHF, n(%) | 91 ( 3) | 67 ( 3) | 24 ( 2) | 0.504 |
| Cardiac arrhythmias, n(%) | 1124 (34) | 777 (34) | 347 (35) | 0.507 |
| Liver disease, n (%) | 780 (24) | 548 (24) | 232 (24) | 0.843 |
| **Severity Score** |  |  |  |  |
| SOFA | 7.00 (5.00, 10.00) | 7.00 (5.00, 10.00) | 7.00 (5.00, 10.00) | 0.556 |
| LODS | 6.00 (4.00, 8.00) | 6.00 (4.00, 8.00) | 6.00 (4.00, 8.00) | 0.467 |
| SAPS II | 44.00 (35.00, 55.00) | 44.00 (35.00, 55.00) | 44.00 (35.00, 55.00) | 0.557 |
| SIC score |  |  |  | 0.5 |
| 4 | 795 (24) | 569 (25) | 226 (23) |  |
| 5 | 1348 (41) | 936 (41) | 412 (42) |  |
| 6 | 1137 (35) | 788 (34) | 349 (35) |  |
| **Vital signs**^a^ |  |  |  |  |
| Mean heartrate, (min^−1^) | 88.93 (79.08, 101.47) | 88.69 (79.26, 101.05) | 89.37 (78.67, 102.62) | 0.658 |
| MAP, (mmHg) | 74.18 (68.29, 80.07) | 74.10 (68.06, 80.09) | 74.26 (68.67, 80.04) | 0.558 |
| Mean resprate, (min^−1^) | 19.62 (16.95, 22.75) | 19.62 (16.89, 22.68) | 19.62 (17.00, 22.87) | 0.804 |
| Mean temperature, (℃) | 36.83 (36.40, 37.25) | 36.83 (36.41, 37.25) | 36.82 (36.37, 37.28) | 0.703 |
| **Laboratory tests**^b^ |  |  |  |  |
| Mean glucose, (mg/dl) | 135.27 (112.49, 158.76) | 135.20 (112.60, 158.67) | 135.43 (112.00, 158.83) | 0.742 |
| Aniongap_max | 16.00 (14.00, 20.00) | 16.00 (14.00, 20.00) | 16.00 (13.00, 20.00) | 0.26 |
| Bicarbonate_min (mEq/L) | 20.00 (17.00, 23.00) | 20.00 (17.00, 23.00) | 20.00 (17.00, 23.00) | 0.74 |
| Chloride_max, (mEq/L) | 109.00 (104.00, 113.00) | 109.00 (104.00, 113.00) | 109.00 (104.00, 113.00) | 0.777 |
| Hematocrit_min, (%) | 26.80 (23.00, 31.10) | 26.90 (23.00, 31.00) | 26.60 (22.70, 31.50) | 0.42 |
| Hemoglobin_min, (g/dL) | 9.10 (7.90, 10.60) | 9.20 (7.90, 10.60) | 9.10 (7.70, 10.60) | 0.474 |
| Lactate_max, (mmol/L) | 3.00 (2.20, 4.80) | 3.00 (2.20, 4.90) | 2.90 (2.13, 4.70) | 0.353 |
| Lowest platelet level, (K/uL) | 93.00 (60.00, 121.00) | 92.00 (58.00, 121.00) | 93.00 (62.00, 122.00) | 0.152 |
| Potassium_max, (K/uL) | 4.60 (4.10, 5.30) | 4.60 (4.10, 5.30) | 4.50 (4.10, 5.20) | 0.126 |
| PTT_max, (s) | 40.70 (32.90, 58.82) | 40.90 (32.90, 59.60) | 40.20 (32.90, 56.90) | 0.382 |
| INR_max, | 1.64 (1.40, 2.20) | 1.60 (1.40, 2.20) | 1.70 (1.40, 2.30) | 0.061 |
| PT_max, (s) | 16.90 (15.00, 21.00) | 16.90 (14.90, 20.90) | 17.00 (15.10, 21.50) | 0.132 |
| Sodium_min, (mEq/L) | 136.00 (133.00, 139.00) | 136.00 (133.00, 139.00) | 136.00 (133.00, 139.00) | 0.193 |
| BUN_max, (mg/dL) | 30.50 (19.00, 50.00) | 31.00 (19.00, 50.00) | 30.00 (19.00, 48.00) | 0.199 |
| WBC_max, (K/uL) | 11.90 (7.70, 17.60) | 12.10 (7.80, 17.60) | 11.50 (7.50, 17.35) | 0.211 |
| Po2-min, (mmHg) | 86.48 (67.00, 102.12) | 86.00 (66.00, 102.20) | 87.00 (67.00, 101.99) | 0.408 |
| Pco2-max, (mmHg) | 45.45 (39.00, 50.00) | 45.48 (39.00, 50.00) | 45.32 (38.00, 50.00) | 0.243 |
| PH-min | 7.31 (7.23, 7.36) | 7.31 (7.23, 7.36) | 7.31 (7.24, 7.37) | 0.047 |
| MCH_min, (pg) | 30.50 (29.30, 32.10) | 30.40 (29.30, 32.10) | 30.60 (29.40, 32.00) | 0.358 |
| MCHC_min, (g/L) | 33.50 (32.40, 34.50) | 33.50 (32.40, 34.50) | 33.50 (32.50, 34.60) | 0.383 |
| RDW_max, (%) | 15.80 (14.60, 17.73) | 15.80 (14.60, 17.80) | 15.80 (14.60, 17.70) | 0.694 |
| MCV_min, (fL) | 90.00 (86.00, 95.00) | 90.00 (86.00, 95.00) | 90.00 (86.00, 95.00) | 0.668 |
| Creatinine-max, (μmol/L) | 123.76 (88.40, 212.16) | 123.76 (88.40, 221.00) | 123.76 (79.56, 203.32) | 0.037 |
| **Infection site, n(%)** |  |  |  |  |
| Lung, n(%) | 1085 (33) | 766 (33) | 319 (32) | 0.572 |
| Urea, n(%) | 884 (27) | 627 (27) | 257 (26) | 0.465 |
| Catheter, n(%) | 87 ( 3) | 56 ( 2) | 31 ( 3) | 0.306 |
| Bacteremia, n(%) | 240 ( 7) | 174 ( 8) | 66 ( 7) | 0.403 |
| Septicemic, n(%) | 48 ( 1) | 39 ( 2) | 9 ( 1) | 0.117 |
| **Treatment measures** |  |  |  |  |
| mv, n(%) | 854 (26) | 611 (27) | 243 (25) | 0.242 |
| Epinephrine, n(%) | 173 ( 5) | 122 ( 5) | 51 ( 5) | 0.924 |
| Norepinephrine, n(%) | 857 (26) | 607 (26) | 250 (25) | 0.522 |

Categorical data were presented as frequency (percentage), parametric continuous data were presented as median (interquartile ranges), whereas non-parametric continuous data were presented as median (interquartile ranges);

^a^Vital signs were calculated as mean value during the first 24 h since ICU admission of each included patients;

^b^The laboratory tests recorded the worest value during the first 24 h since ICU admission of each included patients;

CCU coronary care unit, CSRU cardiac surgical intensive care unit, MICU medical intensive care unit, SICU surgical intensive care unit, TSICU trauma/surgical intensive care unit, SOFA Sequential Organ Failure Assessment, LODS Logistic Organ Dysfunction System, SAPS II Simplified acute physiology II, PT Prothrombin Time, PTT Partial Thromboplastin Time, INR International Normalized Ratio, RDW Red Blood Cell Distribution Widths, MV Mechanical Ventilation, MAP Mean arterial pressure
