# Supplementary material for: Development of a Nomogram to Predict 28-Day Mortality of Patients With Sepsis-Induced Coagulopathy: An Analysis of the MIMIC-III Database
Source: Front Med (Lausanne). 2021 Apr 6;8:661710. doi: 10.3389/fmed.2021.661710 (PMC8056034; doi:10.3389/fmed.2021.661710)
Supplement: Supplementary Table 2 — Accuracy of the nomogram for predicting the risk of 28-day mortality in SIC patients. [file Table_2.DOC]

Additional file Table 2 Accuracy of the Nomogram for predicting the risk of 28-day mortality in SIC patients.

| Variable | Value(95% CI) | |
| --- | --- | --- |
|  | Training set | Testing set |
| AUROC | 0.78(0.74, 0.80) | 0.81(0.78, 0.84) |
| Sensitivity, % | 0.70(0.67, 0.73) | 0.78(0.74, 0.83) |
| Specificity, % | 0.74(0.71, 0.76) | 0.69(0.65, 0.72) |
| Positive predictive value, % | 0.58(0.55, 0.62) | 0.56(0.52, 0.63) |
| Negative predictive value, % | 0.83(0.80, 0.84) | 0.86(0.83, 0.88) |

AUROC area under the receiver operating characteristic curve
